# Feasibility of purely laparoscopic resection of locally advanced rectal cancer in obese patients

**DOI:** 10.1186/1477-7819-10-147

**Published:** 2012-07-16

**Authors:** Tolutope Oyasiji, Keith Baldwin, Steven C Katz, N Joseph Espat, Ponnandai Somasundar

**Affiliations:** 1 Division of Surgical Oncology, Department of Surgery, Roger Williams Medical Center, Boston University, Prior 4, 825 Chalkstone Avenue, Providence, RI, 02908, USA; 2 Department of Surgery Hospital of Saint Raphael, Affiliate of Yale School of Medicine, 1450 Chapel Street, New Haven, CT, 06511, USA

**Keywords:** Laparoscopic, Resection, Advanced, Rectal, Cancer, Obesity

## Abstract

**Background:**

Totally laparoscopic (without hand-assist) resection for rectal cancer continues to evolve, and both obesity and locally advanced disease are perceived to add to the complexity of these procedures. There is a paucity of data on the impact of obesity on perioperative and oncologic outcomes for totally-laparoscopic rectal cancer resection (TLRR) for locally advanced disease.

**Methods:**

In order to identify potential limitations of TLRR, a single-institution database was queried and identified 26 patients that underwent TLRR for locally advanced rectal cancers (T3/T4) over a three-year period. Patients were classified as normal-weight (NW, body mass index (BMI)=18.5 to 24.9kg/m2), overweight (OW, BMI=25 to 29.9kg/m2) and obese (OB, BMI >/= 30kg/m2). Perioperative outcomes, lymph node harvest and margin status were assessed.

**Results:**

Seven patients were classified as NW (26.9%), 12 as OW (46.2%) and 7 as OB (26.9%). Age, tumor stage, gender and American Society of Anesthesiologists (ASA) scores were similar. OB had more co-morbidities (median 3.0, range 0.0 to 5.0 vs. 2.0, range 0.0 to 3.0 for NW and 1.0, range 0.0 to 3.0 for OW). Five patients had tumors <5cm from anal verge (NW=2; OW=1; OB=2). A median of 19.0, range 9.0 to 32.0; 20.0, range 9.0 to 46.0 and 19.0, range 15.0 to 31.0 lymph nodes were retrieved in the NW, OW and OB, respectively (Not Significant (NS)). Median node ratios for NW, OW and OB were 0.32, 0.13 and 0.00, respectively. All groups had negative proximal and distal margins. Radial margins were negative for 100% of NW, 83.3% of OW and 85.7% of OB (NS). Conversion rates were 14.3% for NW, 16.7% for OW & 0% for OB (NS). NW, OW and OB had complication rates of 28.3%, 33.3% and 14.3%, respectively. Median operative time, median estimated blood loss and median length of hospital stay were similar for all groups.

**Conclusion:**

The perceived limitation that obesity would have on TLRR was not demonstrated by the analyzed data. Although our findings are limited by the modest sized cohort, the results suggest that it is reasonable to offer TLRR to obese patients with rectal cancer.

## Background

The introduction of laparoscopic rectal cancer surgery raised concerns about the feasibility, safety and oncologic adequacy of this modality. In recent years, the laparoscopic approach has come to be accepted as technically feasible and safe [1-4], with the TLRR gaining increasing acceptance.

The incidence of obesity is rising globally with 11% and 20% of the populations in France and the United States, respectively, reported as obese [1]. It is clear that the challenging situation of applying TLRR to obese patients is being encountered more frequently. In the recent past, the presence of locally advanced disease was considered a contraindication to TLRR. However, the modality is being increasingly used as locally advanced disease in obese patients is encountered more frequently [1].

Obese patients undergoing laparoscopic colorectal surgery present a technical challenge. Blood loss, duration of surgery and conversion rates have been reported to be greater for obese patients compared to the non-obese (1, 3 and 4). However, morbidity and mortality are comparable for both groups [1,3,4]. Adequacy of oncologic resections has also been verified in laparoscopic colon cancer resection [1,2]. However, both benign and malignant colonic conditions are included in most studies and few reports focus exclusively on laparoscopic colectomy for cancer [1,3-6]. In some cases colon and rectal cancers have been combined, and very few studies have evaluated the impact of obesity on TLRR [1,7]. Rather than compare outcomes of purely laparoscopic technique for rectal cancer resection between obese and non-obese patients, some series have mixed up cases of hand-assisted laparoscopic and purely laparoscopic techniques, in drawing comparison between the two groups. As such, there is insufficient broadly applicable data to guide the selection of obese patients for TLRR in the management of locally advanced rectal adenocarcinoma.

The goal of this study was to analyze the impact of obesity on perioperative outcomes for TLRR surgery done for locally advanced rectal cancer. We also assessed the lymph node retrieval and margin status of these operations as surrogates for oncologic adequacy.

## Methods

Between July 2007 and March 2010, 26 patients with locally advanced rectal cancer (T3 or T4) underwent resection using TLRR. Preoperative staging included endorectal ultrasonography, thoracoabdominopelvic computerized tomographic scan or pelvic magnetic resonance imaging. After obtaining Institutional Review Board approval, a prospectively maintained database was queried for demographic, oncologic, perioperative, morbidity, mortality and pathologic data. Data collected included age, gender, body mass index (BMI), preoperative diagnosis, neoadjuvant therapy, distance to anal verge, co-morbidities, type of operation, ASA score, operative time, estimated blood loss, type of operation, intraoperative and postoperative complications, length of hospital stay, conversion rates, number of lymph nodes retrieved and margin status. Patients were categorized as normal weight (BMI 18.5 to 24.9kg/m^2^), overweight (BMI 25 to 29.9kg/m^2^) and obese (BMI >/= 30kg/m^2^). NW, OW and OB were compared to identify potential differences in operating time, estimated blood loss, length of stay, complication, conversion, lymph node retrieval and margin status.

### Technique

Four ports were routinely used, with placement in the left upper, right upper, right lower and suprapubic positions. Occasionally a fifth port in the left lower quadrant was inserted to facilitate retraction of the colon when a bulky mesentery was encountered, particularly in obese patients. Lesions were confirmed by rectal or proctoscopic examination, as well as preoperative tattooing. The medial to lateral approach was utilized for our dissection and mobilization. The inferior mesenteric artery (IMA) was dissected and staple divided. The left ureter was identified, and the prescaral plane was carefully developed with preservation of the autonomic nerve fibers using the technique of sharp total mesorectal excision. The rectum was divided at least 3cm distal to the tattoo using an articulating stapling device via the suprapubic port. The splenic flexure was mobilized as deemed necessary. A small 4 to 5cm incision was then made for specimen extraction and proximal transection, and a circular stapler was used for all anastomoses.

## Results

### Demographics

Twenty-six total patients; 15 male (57.7%) and 11 female (42.3%) with T3 or T4 rectal cancers are reported in this series (Table1). They were categorized into NW (n=7 (26.9%)), OW (n=12 (46.2%)) and OB (n=7 (26.9%)). The median ages (NW=65yr, OW=63yr, and OB=66yr) were also similar (Table1). The median BMIs for the three groups were 20.9kg/m^2^ (NW), 28.2kg/m^2^ (OW) and 36.1kg/m^2^ (OB).

**Table 1:** Demographics and other characteristics

**Characteristic**	**Normal weight**	**Overweight**	**Obese N=7**
	**N=7**	**N=12**	
Sex			
Male; no (%)	5 (71.4%)	8 (66.7%)	2 (28.6%)
Female; no (%)	2 (28.6%)	4 (33.3%)	5 (71.4%)
Median age (yr)	65	63	66
Range (yr)	52 to 85	46 to 86	52 to 76
Median BMI(kg/m^2^)	20.9	28.2	36.1
Range (kg/m^2^)	18.8 to 24.9	25.5 to 29.8	31.1 to 41.5
Median no. of comorbidities	2.0	1.0	3.0
Range	0.0 to 3.0	0.0 to 3.0	0.0 to 5.0
Median ASA score	2.0	2.0	3.0
Range	2.0 to 4.0	2.0 to 3.0	2.0 to 4.0

Altogether, there were 22 (84.6%) T3 tumors and 4 (15.4%) T4 tumors. There was a comparable distribution of tumor stages across the three BMI groups (Table2). A total of 77% of patients (n=20) had rectal cancer while 23% (n=6) had rectosigmoid cancers. The rectosigmoid cancers were preoperatively and intraoperatively identified and treated as cancers in the upper third of the rectum. In the NW group, 57.1% underwent neoadjuvant therapy; as opposed to 16.7% of OW patients and none of the OB patients. The presence of co-morbidities, including coronary artery disease, hypertension, diabetes mellitus, hyperlipidemia, chronic kidney disease, acute kidney disease, asthma, chronic obstructive pulmonary disease, hypothyroidism and hyperthyroidism, was documented for each patient. In terms of medical co-morbidities, NW patients had a median of 2.0 co-morbidities, compared to 1.0 and 3.0 in the OW and OB groups. The median ASA scores were 2.0, 2.0, and 3.0 respectively.

**Table 2:** Tumor characteristics and distribution

**Characteristic**	**Normal weight**	**Overweight**	**Obese**
T stage			
T3	6	9	7
T4	1	3	0
Neoadjuvant treatment	4	2	0
Very Low Tumor (<5cm from anal verge)	2	1	2

Very low rectal tumors were defined as those that are less than 5cm from the anal verge. Distance from the lower edge of the tumor to the anal verge was recorded for the patients. A total of five patients had very low tumors (NW=2; OW=1; OB=2).

### Perioperative outcomes

The median operative times for the NW, OW and OB groups were 195.0 minutes; 240.5 minutes and 264.0 minutes, respectively. There were no significant differences among the groups with respect to length of stay or blood loss (Table3). The rates of conversion to open procedure were comparable for the NW and OW groups at 14.3% and 16.7%, respectively, while the OB group had no conversions. Major and minor complications occurred in 28.3% of NW, 33.3% of OW and 14.3% of OB (Tables3 and 4). For the purpose of standardization, the complications were also graded using the Clavien Score. The only mortality in the series was recorded in the OW group as a result of postoperative massive pulmonary embolism in a patient who underwent laparoscopic abdominoperineal resection for T3 rectal cancer. This patient had neoadjuvant chemoradiation therapy.

**Table 3:** Outcomes

**Parameter**	**Normal weight**	**Overweight**	**Obese**
	**N=7**	**N=12**	**N=7**
Median LOS (days)	12.0	7.0	5.0
Range	4.0 to 30.0	3.0 to 14.0	4.0 to 17.0
Median estimated blood loss (ml)	200.0	200.0	200.0
Range	50.0 to 700.0	50.0 to 650.0	125.0 to 500.0
Median operative time (minutes)	195.0	240.5	264.0
Range	125.0 to 351.0	127.0 to 578.0	143.0 to 359.0
Median number of lymph nodes retrieved	19.0	20.0	19.0
Range	9.0 to 32.0	9.0 to 46.0	15.0 to 31.0
Median lymph node ratio (positive/total)	0.32	0.13	0.00
Range	0.00 to 0.72	0.00 to 0.61	0.00 to 0.83
Negative margins (radial)	7 (100%)	10 (83.3%)	6(85.7%)
Negative margins (proximal/distal)	7 ([100%)	12 (100%)	7 (100%)
Conversion	1 (14.3%)	2 (16.7%)	0 (0%)
No. of patients with complications	2 (28.3%)	4 (33.3%)	1 (14.3%)
Mortality	-	1	-

**Table 4:** Complications

**Complication**	**Clavien Score**	**Normal weight**	**Overweight**	**Obese**
		**(n=7)**	**(n=12)**	**(n=7)**
Sacral decubitus ulcer	Grade I	1	-	-
Acute renal failure	Grade I	1	-	-
Surgical site infection	Grade I	1	-	2
Ileus	Grade I	1	-	1
Pulmonary embolism	Grade II	1	1	-
Pneumonia	Grade II	2	-	-
UTI	Grade II	1	1	-
Pelvic abscess	Grade IIIa	2	-	-
Anastomotic leak	Grade IIIb	1	-	-
Bleeding requiring reoperation	Grade IIIb	1	-	-

### Oncologic parameters

The three groups were compared for lymph node retrieval on pathologic examination of resected specimens, as well as proximal, distal and radial margins. The median lymph node harvest was 19.0 for the NW group, 20.0 for the OW group, and 19.0 for the OB group. Median node ratios for NW, OW and OB were 0.32, 0.13 and 0.00, respectively. All patients in the study had negative proximal and distal bowel margins. Radial margin negativity rates were 100%, 83.3% and 85.7% for NW, OW and OB, respectively.

## Discussion

There is a paucity of data on the relationship between obesity and outcomes of totally laparoscopic resections for rectal cancer (TLRR). Bege *et al.* were the first to compare outcomes of laparoscopic rectal resection for cancer between obese and non-obese patients. They reported increased conversion rate and operative time for obese patients but the overall morbidity was comparable for both groups [1]. The bulk of data on the impact of obesity on colorectal surgery is predominantly from colonic resections. Results have been conflicting, with Delaney *et al.* reporting no difference in operative time, complication rate or cost [2] while other groups reported significant differences in those parameters [3,4].

Laparoscopic rectal resection for malignancy is technically more demanding. This may at least partly explain the limited availability of this procedure and lack of relevant data. Concerns about nerve preservation, complete total mesorectal excision, and adequate lymph node harvest are still unanswered in this setting.

In view of the peculiar technical challenges of laparoscopic rectal cancer surgery, it would be inappropriate to simply extrapolate the data from laparoscopic colonic resections [1]. The CLASSIC trials by Guillou *et al.* in 2005 and 2007 are Level 1 studies that addressed short-term and long-term outcomes of laparoscopic assisted colorectal cancer surgery [5,6].

TLRR is evolving. This unique study specifically addresses a TLRR with emphasis on locally-advanced disease defined as T3/T4. Additionally, the role of varying levels of obesity is examined. Besides the association between obesity and occurrence of colorectal cancer [8], the safety, feasibility, short-term outcomes and oncologic effectiveness of this approach could be affected by obesity. This was examined in the present study. Median age, gender distribution, cancer location, tumor stage, medical comorbidities, ASA score, lymph node harvest and margin positivity rates were comparable for the three BMI groups studied.

The presence of lymph node metastasis is important for predicting the clinical outcome of patients who have undergone radical surgery for colorectal carcinoma and for making decision on need for adjuvant therapy. Therefore, an accurate measurement of the pathologic status of the tumor lymph nodes in the specimen is important for reducing the risk of understaging [9]. This study showed no difference in the median number of lymph nodes harvested in each of the three weight groups (NW=19.0, range 9.0 to 32.0; OW=20.0, range 9.0 to 46.0; OB=19.0, range 15.0 to 31.0). Median node ratios for NW, OW and OB (0.32, range 0.00 to 0.72; 0.13, range 0.00 to 0.61 and 0.00, range 0.00 to 0.83 respectively) also did not show any significant difference. Although the AJCC and IUAC guidelines [10] suggest >12 lymph nodes as an adequate harvest for colon cancer, it is unclear whether these node thresholds should apply to rectal cancer especially for patients who had neoadjuvant treatment. Nonetheless, in the present study of the laparoscopic technique, the average nodal harvest was well in excess of these recommendations. However, Gorog *et al.* reported a finding of significantly lower number of retrieved lymph nodes from rectal resection specimens in patients with BMI >/= 25kg/m^2^ compared to those with BMI<25kg/m^2^ when the length of the resected specimen was less than 16cm [11]. These findings indicate that the quantity and adequacy of mesorectal lymph node dissection for rectal cancer has not been firmly established.

Negative proximal/distal margin rates were 100% for the NW, OW and OB groups. Negative radial margin rates were 100%, 83.3% and 85.7% for the NW, OW and OB groups respectively. The two patients in the OW group with positive radial margin presented with obstructing tumors which required prompt surgical intervention that made it impossible for them to undergo neoadjuvant chemoradiation. Both were extraluminal, invasive rectal tumors with adherence to surrounding structures. The only patient in the OB group with positive radial margin presented with bleeding rectal tumor necessitating prompt surgery, which also prevented administration of neoadjuvant chemoradiation.

Operative time increased slightly with progression from non-obese to obese patients, but was not significant. The small numbers in this study certainly do not contradict the findings from other studies that have documented longer duration of surgery for obese patients [1]. Median blood loss was similar for the three groups, standing at 200ml.

The rate of complications for the OB group (14.3%) is almost half of that for NW (28.6%) and OW (33.3%)-an unusual observation. This may be explained by the fact that none of the patients in the OB group underwent neoadjuvant chemoradiotherapy compared to 57.1% of NW and 16.7% of OW. This observation is congruent with earlier studies which have associated neodjuvant chemoradiation with greater technical difficulty and increased perineal wound complications in laparoscopic rectal cancer resection [12]. For ease of universal reference and standardization of documentation, each complication was graded using the Clavien Score.

The overall conversion rate in this series of locally-advanced rectal cancers (T3/T4) is 11.5%. Conversion rate for laparoscopic rectal cancer resection is documented in the Conventional versus Laparoscopic-Assisted Surgery in Colorectal Cancer [CLASICC] trial to be 34%. More recently it has been documented in the literature as ranging from 2.8 to 9.8% [13-16], for all T-stages combined. The authors’ experience with lower conversion rates in locally advanced tumors likely reflects the continual refinement that laparoscopic rectal surgery has undergone since the CLASICC trial, in addition to careful patient selection.

In this study, the NW group had a longer median length of hospital stay than the OW and OB groups. The presence of an outlier in the NW group with length of stay of 30days is noted. The postoperative course in this patient was complicated by prolonged ileus initially, followed by rapid atrial fibrillation, fungemia, pneumonia and superficial wound infection.

Over the years concerns have arisen regarding the results of laparoscopic rectal cancer resection compared with open conventional resection. Many studies have documented longer operative time for laparoscopic rectal cancer resection compared to the open approach. However, this shortcoming is often offset by shorter hospital stays, earlier return of bowel function, decreased blood loss and better preservation of autonomic nerves [17,18]. In terms of five-year local and distant recurrence rates, five-year overall survival and disease-free survival, results are comparable, without the benefit of large series [14-16,19,20].

## Conclusion

The present series adds to the body of literature supporting the feasibility and safety of the totally laparoscopic technique in treating obese patients with locally advanced (T3/T4) rectal cancers which could also be confirmed with further studies. Both perioperative outcomes and surrogates of oncologic surgical quality from TLRR are comparable to the reported results for non-obese patients in the current literature. While this study is limited by a modest cohort, the data support that totally laparoscopic rectal resection may be an appropriate and safe technique applicable to obese patients.

## Abbreviations

BMI, Body mass index; IMA, Inferior mesenteric artery; NS, Not significant; NW, Normal weight; OW, Overweight; OB, Obese; TLRR, Totally-laparoscopic rectal cancer resection.

## Competing interests

There are no competing interests and we have not received any grants or financial support for this study.

## Authors’ contributions

TO, KB, SK, JE and PS made substantial contributions to the concept and design of the research, data acquisition and analysis, manuscript drafting and revision, as well as final approval for publication. All authors read and approved the final manuscript.
